# Complete genome sequence of *Ostreid herpesvirus-1* associated with mortalities of *Scapharca broughtonii* broodstocks

**DOI:** 10.1186/s12985-015-0334-0

**Published:** 2015-07-25

**Authors:** Junyang Xia, Changming Bai, Chongming Wang, Xiaoling Song, Jie Huang

**Affiliations:** College of Marine Life Sciences, Ocean University of China, Qingdao, China; Key Laboratory of Sustainable Development of Marine Fisheries, Ministry of Agriculture, Yellow Sea Fisheries Research Institute, Chinese Academy of Fishery Sciences, Qingdao, China; Function Laboratory for Marine Fisheries Science and Food Production Processes, Qingdao National Oceanography Laboratory, Qingdao, China

**Keywords:** Herpesvirus, OsHV-1, Bivalves, Mortality, Genome sequence

## Abstract

**Background:**

*Ostreid herpesvirus-1* (OsHV-1) is the major bivalve pathogen associated with severe mortality events in a wide host range. In the early summer of 2012 and 2013, mass mortalities of blood clam (*Scapharca broughtonii*) broodstocks associated with a newly described variant of OsHV-1 (OsHV-1-SB) were reported.

**Methods:**

In this study, the complete genome sequence of the newly described variant was determined through the primer walking approach, and compared with those of the other two OsHV-1 variants.

**Results:**

OsHV-1-SB genome was found to contain 199, 354 bp nucleotides with 38.5 % G/C content, which is highly similar to those of acute viral necrosis virus (AVNV) and OsHV-1 reference type. A total of 123 open reading frames (ORFs) putatively encoding functional proteins were identified; eight of which were duplicated in the major repeat elements of the genome. The genomic organization of OsHV-1-SB could be represented as TR_L_-U_L_-IR_L_-IR_S_-U_S_-TR_S_, which is different from that of OsHV-1 reference type and AVNV due to the deletion of a unique region (X, 1.5Kb) between IR_L_ and IR_S_. The DNA sequence of OsHV-1-SB is 95.2 % and 97.3 % identical to that of OsHV-1 reference type and AVNV respectively. On the basis of nucleotide sequences of 32 ORFs in OsHV-1-SB and the other nine OsHV-1 variants, results from phylogenetic analysis also demonstrated that OsHV-1-SB is most closely related to AVNV.

**Conclusions:**

The determination of the genome of OsHV-1 with distinguished epidemiological features will aid in our better understanding of OsHV-1 diversity, and facilitate further research on the origin, evolution, and epidemiology of the virus.

**Electronic supplementary material:**

The online version of this article (doi:10.1186/s12985-015-0334-0) contains supplementary material, which is available to authorized users.

## Background

Since the first reported mortality associated with a herpes-like virus in Eastern oyster (*Crassostrea virginica*) in 1972 [1], bivalve herpesvirus infection has been associated with mortalities and heavy losses in cultured bivalves worldwide [2–5]. High mortalities of bivalves associated with bivalve herpesvirus infection were usually reported in spats and juveniles of Pacific oyster (*Crassostrea gigas*) [6–9]. These mortalities were usually related to an increase in temperature of sea water, but different from previously described “summer mortality” of *C. gigas* adults during the summer months [10, 11]. From 2008 in France, more severe mortalities with mortality rates averaging 80 % were reported mainly affecting spats and juveniles [10–13]. Mass mortalities of bivalves associated with herpes-like virus (referred to hereafter as Acute viral necrosis virus, AVNV) were also reported during the summer months in China in 1990s [14]. The disease has also occurred annually in the summer in China, with mortalities reached more than 90 % within 5–8 days after first appearance [15, 16]. However, mass mortalities of bivalves associated with AVNV infection in China were usually found in Chinese scallop (*Chlamys farreri*) adults [14, 17].

The genomes of two herpesvirues purified from bivalves have been completely sequenced [18, 19]. The first sequenced genome was obtained using viral particles purified from moribund Pacific oyster larvae (GenBank number AY509253) [18], which allowed the virus to be assigned as the founding member of the species *Ostreid herpesvirus 1* (OsHV-1), genus *Ostreavirus*, family *Malacoherpesviridae* [20]. Then the completion of the genomic sequence of AVNV (GenBank number GQ153938) and comparison with that of OsHV-1 indicated that AVNV is a variant of OsHV-1 [19]. Based on nucleotide differences of partial genomes, several further variants of OsHV-1 were also identified associated with epidemic or sporadic mortalities of bivalves [21, 6, 12, 22]. Compared to OsHV-1 reference type, the occurrence of more virulent OsHV-1 variants has been reported in larvae and spat [12, 13], and the reported temperature thresholds associated the onset of mortalities have decreased from 19 °C to 16 °C [23].

Blood clam (*Scapharca broughtonii*) was one of the most commercially important shellfish cultivated in China. The development and rapid expansion of intensive farming system have been accompanied by the occurrence of several threatening diseases [24, 25]. During the early summer of 2012 to 2013, mass mortalities of blood clam brood stocks were reported in several hatcheries in the north coast of China. Enveloped herpesvirus-like particles were found within the digestive glands, gills and mantles of moribund blood clams collected in 2012 and 2013 by transmission electron microscopy. Quantitative PCR analysis adapted from a previously published protocol [26], also indicated the presence of high levels (7.06 × 10^3^ to 2.58 × 10^7^ copies mg^−1^ of tissue) of OsHV-1 DNA in these samples (unpublished data). The variant infected and associated with the mortalities of *S. broughtonii* in China was named as SB variant of OsHV-1 (referred to hereafter as OsHV-1-SB).

Genome sequencing of multiple variants of OsHV-1 with distinguished characteristics of epidemiology will provide rich data on variations among these variants at both the DNA and amino acid levels, which will subsequently facilitate further research on the origin, evolution, and epidemiology of the virus. OsHV-1-SB was the first variant that found to infect and associate with mass mortalities in Arcidae bivalve mollusks around the world. In this report, we sequenced the complete DNA sequence of virus particles purified from moribund blood clams collected in 2012. The coding capacity and genetic content of the OsHV-1-SB genome were analyzed and compared to that of OsHV-1 reference type and AVNV.

## Results and discussion

### General characteristics of the OsHV-1-SB genome

To understand the genetic variations of OsHV-1 infecting different bivalve species, the sequence of viral particles purified from moribund blood clams was determined. About 6× coverage of the OsHV-1-SB genome was accomplished. The resolved OsHV-1-SB genome sequence was found to be a double-stranded DNA with 199,354 bp in length, which is 8, 085 bp and 11, 639 bp shorter than that of OsHV-1 reference type and AVNV, respectively. The nucleotide sequence of OsHV-1-SB genome is 95.2 % and 97.3 % identical to that of OsHV-1 reference type and AVNV. The overall nucleotides content of G + C composition is 38.5 %, which is identical to that of AVNV and similar to that of OsHV-1 reference type (38.7 %). As for the genome structure, OsHV-1-SB is similar to OsHV-1 reference type and AVNV, which consists of two unique regions (U_L_ and U_S_; 172.7 kb and 4.6 kb, respectively), each flanked by an inverted repeat (TR_L_/IR_L_ and TR_S_/IR_S_; 3.6 and 7.5 kb, respectively). However, different from the genomic structure of OsHV-1 reference type and AVNV, the third unique region (X, 1.5 Kb) between IR_L_ and IR_S_ was deleted in that of OsHV-1-SB. Thus, the genomic arrangement of OsHV-1-SB could be represented as TR_L_-U_L_-IR_L_-IR_S_-U_S_-TR_S_, which is a typical structure of D-type herpesviral genome [27]. Davison et al. [18] have already reported a small proportion of molecules in OsHV-1 reference type DNA may also lack the third unique region (X) as found in OsHV-1-SB.

**Fig. 1:**
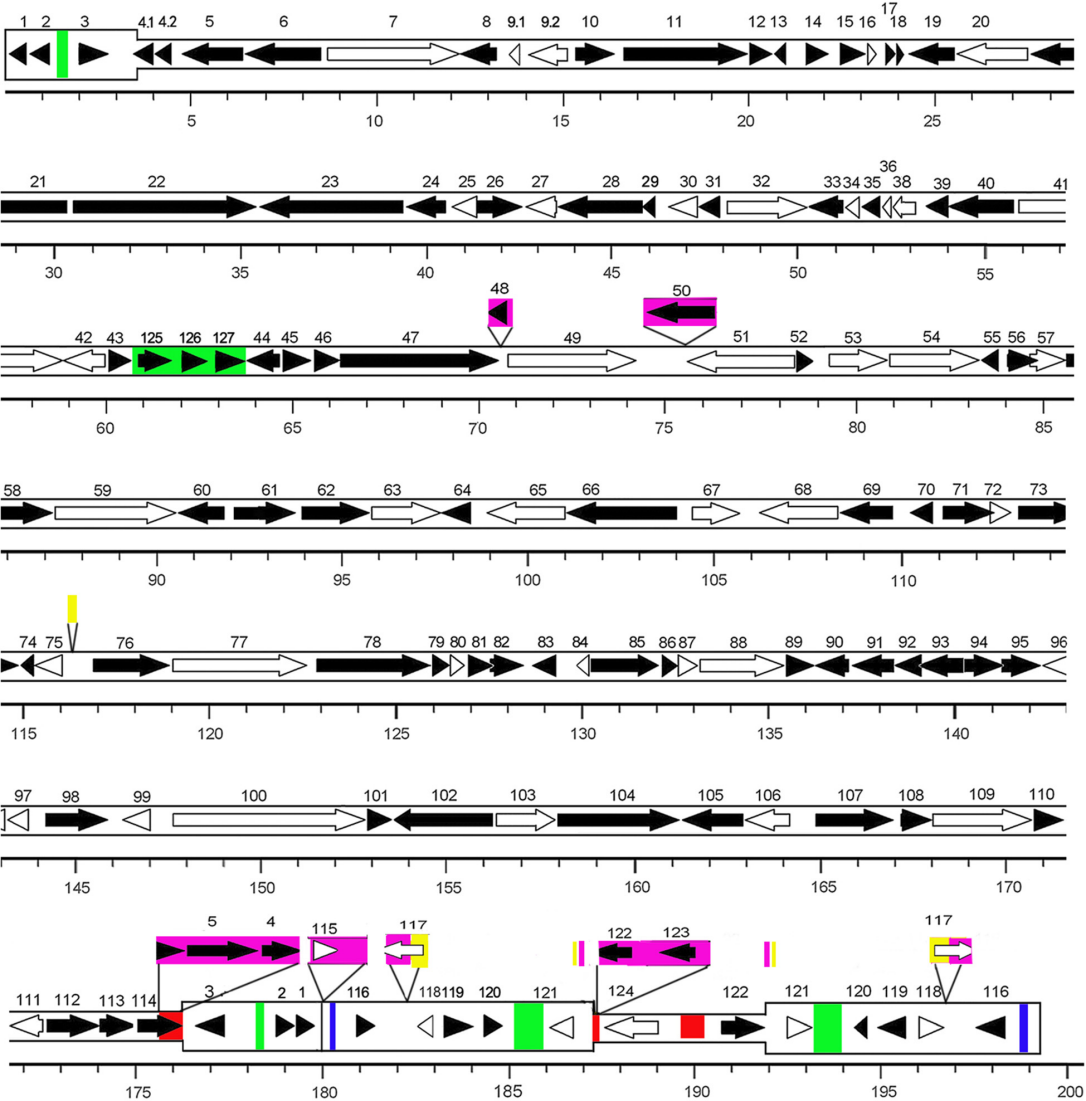
Layout of ORFs in the OsHV-1-SB genome. The inverted repeats TRL/IRL (ORF1-ORF3) and TRS/IRS (ORF116-ORF121) were shown in a thicker format. White arrows indicated ORFs with putative functions similar to its counterparts in OsHV-1 reference type and AVNV. Black arrows indicated ORFs with unknown function. Red rectangles indicated insertions found only in OsHV-1-SB; green rectangles indicated insertions found both in OsHV-1-SB and AVNV; blue rectangles indicated insertions found both in OsHV-1-SB and OsHV-1 reference type; purple rectangles indicated deletion found only in OsHV-1-SB; yellow rectangles indicated deletion found both in OsHV-1-SB and AVNV

The genome was predicted to encode 123 unique ORFs, ranging from 71 to 1,878 amino acid residues in length. Eight of the 123 ORFs were duplicated within the inverted repeats, and resulting in a total of 131 putative genes in the genome (Additional file 1: Table S1, Fig. 1). The nomenclature of these ORFs was according to the OsHV-1 reference type genome. OsHV-1-SB ORFs that have counterparts in OsHV-1 reference type were given the same names as OsHV-1 reference type (ORFs 1–124), and ORFs that lack counterparts were designated by new names (ORFs 125–127). 96 and 94 predicted ORFs in OsHV-1-SB were conserved (96–100 % identity) to its counterparts in OsHV-1 reference type and AVNV respectively, and eleven of them (ORFs 2, 13, 30, 35, 36, 52, 74, 81, 91, 96 and 109) were completely identical among all three variants. Of the eleven highly conserved ORFs in OsHV-1, ORF 30 and ORF 109 also have homologues in vertebrate herpesviruses [18]. And they perhaps were the only two ORFs in OsHV-1 supporting a common ancestry for OsHV-1 and the other herpesvirus [18].

### Genetic variations due to indels

As a result of indels occurred in OsHV-1 genome, many gaps are created in OsHV-1-SB genome relative to OsHV-1 reference type and AVNV, due to which a number of ORFs were found to be deleted, inserted or rearranged. According to the results of genome comparison, eight large insertions (>63 bp) and twelve large deletions (>154 bp) were recognized in OsHV-1-SB respectively. Among the top eight largest insertions, only one insertion (2.6 Kb, between 60,819 bp and 63,478 bp) was predicted to encode three new ORFs (ORFs 125, 126 and 127) with unknown function. This insertion was also present in AVNV, but was not annotated by Ren et al. [19]. Of the twelve largest deletions, seven of them resulted in the deletions of nine ORFs (ORFs 4, 5, 48, 50, 115, 117 both in IR_S_ and TR_S_, 122 and 123) and rearrangement of ORF 114 in OsHV-1-SB (Table 1). Some of the deleted ORFs in OsHV-1-SB were found to belong to special gene families, whose products were predicted to encode membrane-associated proteins (ORF 5), Ring finger proteins (ORF 117), motifs V and VI of SF2 helicases (ORF 115) and secreted proteins (ORF 50) [18]. There were eight ORFs (ORFs 3, 12, 21, 38, 68, 70, 106 and 120) mutated as a result of small indels in OsHV-1-SB (Table 2).

**Table 1:** Large insertions/deletions and associated variations of ORFs in OsHV-1-SB compared to OsHV-1and AVNV

Variation types	Position	Size	Affected regions	Affected ORFs	Status in OsHV-1 and AVNV^a^
Insertion	1654-1741	87	TR_L_	*None*	AVNV
	60819-63478	2659	U_L_	125, 126, 127	AVNV
	178067-178155	88	IR_L_	*None*	AVNV
	180167-180231	64	IR_S_	*None*	OsHV-1
	184924-185970	1046	IR_S_	*None*	AVNV
	189425-190310	885	U_S_	*None*	*None*
	193194-194240	1046	TR_S_	*None*	AVNV
	198929-198993	64	TR_S_	*None*	OsHV-1
	175367-176273	906	U_L_	114	*None*
	187270-187366	96	U_S_	*None*	*None*
Deletion	70706-70707	599	U_L_	48	*None*
	75526-75527	1849	U_L_	50	*None*
	116350-116351	450	U_L_	*None*	AVNV
	179809-179810	1506	IR_S_	115	*None*
	182505-182506	1188	IR_S_	117	AVNV
	186872-186873	115	IR_S_	*None*	AVNV
	187088-187089	235	IR_S_	*None*	*None*
	192054-192055	235	TR_S_	*None*	*None*
	192155-192156	155	TR_S_	*None*	AVNV
	196659-196660	1188	TR_S_	117	*None*
	175367-176273	4113	U_L_	4, 5, 114	*None*
	187270-187366	3368	U_S_	122, 123	*None*

^a^indicated whether the given variation was present in OsHV-1, AVNV or none of them

**Table 2:** Small insertions/deletions and associated variations of ORFs in OsHV-1 variants

ORF	Variants	Positions^a^	Variation of nucleotides	Variation of amino acids
3	OsHV-1-SB	*None*	*GAGG-TATTGCTGCTGCCAGTAACAACACCACC*	*None*
	OsHV-1	28	GAGGGTATTGCTGCTGCCAGTAACAA---CACC	EGIAAASNNT
	AVNV	28	GAGGGTATTGCTGCTGCCAGTAACAA---CACC	EGIAAASNNT
12	OsHV-1-SB	*None*	*GATAA-GGAAGAA*	*None*
	OsHV-1	99	GATAAAGGAAGA	DKGR
	AVNV	99	GATAAAGGAAGA	DKGR
21	OsHV-1-SB	1942	(TTC)_4_TACCAAAATGGTGAGGAGGGTTCA	FFFFYQNGEEGS
	OsHV-1	1942	(TTC)_3_TACCAAAATGGTGAGGAGGGTTCA	FFF-YQNGEEGS
	AVNV	1942	(TTC)_3_TACCAAAATGGTGAGGAGGGT-CAT	FFF-YQNGEEGH
38	OsHV-1-SB	*None*	*AAGCC-ATG*	*None*
	OsHV-1	118	AAGCCCATG	KPM
	AVNV	118	AAGCCCAT-	KPM
38	OsHV-1-SB	398	GATTTTTT--CAC	DFFH
	OsHV-1	524	GATTTTTTT-CAC	DFFT
	AVNV	*None*	*GATTTTTTTTCAC*	*None*
68	OsHV-1-SB	451	GGA(GGT)_6_GGA	GGGGGGGG
	OsHV-1	451	GGA---------(GGT)_3_GGA	G---GGGG
	AVNV	451	GGA------(GGT)_4_GGA	G--GGGGG
70	OsHV-1-SB	472	CAC--TTCAAAAAT	HFKN
	OsHV-1	472	CACACTTCAAAA	HTSK
	AVNV	472	CACACTTCAAAA	HTSK
106	OsHV-1-SB	18	GGGGGGGTTTGTA	GGFV
	OsHV-1	553	GGGGGG-TTTGTA	GGFV
	AVNV	553	GGGGGG-TTTGTA	GGFV
106	OsHV-1-SB	610	G(GAG)_8_TGA	GGGGGGGGV
	OsHV-1	1143	G(GAG)_5_---------TGA	GGGGG---V
	AVNV	1143	G(GAG)_3_------------TGA	GGGG----V
120	OsHV-1-SB	301	AACAGGGGGGAT	NRGD
	OsHV-1	301	AACA-AGGGGAT	NKGI
	AVNV	301	AACGGGGGGGAT	NGGD

^a^ indicated positions of the first nucleotides shown in the next column in the given ORFs

“*None*” indicated the sequences shown in the next column (italic) were not predicted to encode ORFs any more in the variant due to small indels occurred in the given sequences

“-” indicated a deletion of nucleotide or amino acid

**Table 3:** Characterization of SNPs in three OsHV-1 variants

Regions	All three variants		OsHV-1-SB vs OsHV-1		OsHV-1-SB vs AVNV		AVNV vs OsHV-1	
	No. of SNP	Frequency (bp/Kb)	No. of SNP	Frequency (bp/Kb)	No. of SNP	Frequency (bp/Kb)	No. of SNP	Frequency (bp/Kb)
SNPs in genomes	1037	5.4	698	3.6	859	4.5	513	2.6
SNPs in ORFs	727	4.4	498	3	635	3.8	374	2.2
SNPs in non-coding regions	310	11.9	200	7.6	224	8.6	148	5.7
SNPs in fragmented ORFs	69	5.3	42	3.3	60	4.7	35	2.7
SNPs in ORFs (not counting fragmented ORFs)	658	4.3	456	3	575	3.7	339	2.2

In addition, the DNA sequence of one deletion (559 bp) in the U_L_ region was identical to that of an insertion in the Us region of OsHV-1-SB, which may indicated potential recombination has occurred in OsHV-1-SB.

Genome arrangements as a result of indels have also been reported in the other variants of OsHV-1 [28, 13]. For example, ORF48 and ORF114 were found to display similar genetic variations in these variants as those showed in OsHV-1-SB [28]. Rearrangements of ORF35, −38 and deletions of ORF36, −37 have also been reported in OsHV-1 variants detected in France and several other countries since 2008 by Renault et al. [11]. And more severe mortalities of *C. gigas* have been reported associated with infection of these variants compared to that of OsHV-1 reference type [12, 10]. Studies in vertebrate herpesviruses have also found that genome rearrangements as a result of indels were associated with viral replication and pathogenicity [29–31]. For example, different strains of *Human Cytomegalovirus* were found to exhibit different virulence and tissue tropism due to the deletion of some genes after a long-term passages in laboratory [31]. Recently, through the techniques of reverse transcription quantitative PCR, Segarra et al. [32] found the transcripts of ORFs 4, 38, 106 could be detected in *C.gigas* larves at 2 h after experimental infection, and at 4 h for ORF117 [32]. These results suggested an important role of the deleted or rearranged ORFs in virus replication and disease development. Currently, there was little information available about the roles of the deleted or rearranged ORFs in the pathogenicity of OsHV-1 variants.

### Characterization of SNPs

From the whole genome sequences of the three variants, we identified 1037 SNPs. Overall, SNPs were found at the rate of 5.4 per kbp among the genomes of three OsHV-1 variants (Table 3). The SNPs were distributed unevenly across the genomes, which occurred 2.7 times more frequently in non-coding sequences than in coding regions. Additionally, SNP occurred more frequently in fragmented ORFs than in normal ORFs. Despite the highest nucleotide similarity at the genome level and closest phylogenetic relationships displayed between OsHV-1 reference type and AVNV, OsHV-1-SB and AVNV exhibit the highest SNP frequency (4.5 per kbp), followed by OsHV-1-SB and OsHV-1 reference type (3.6 per kbp) and OsHV-1 reference type and AVNV (2.6 per kbp). It is difficult to explain how and why the discordance occurred here based on current results.

### Phylogenetic analysis

**Fig. 2:**
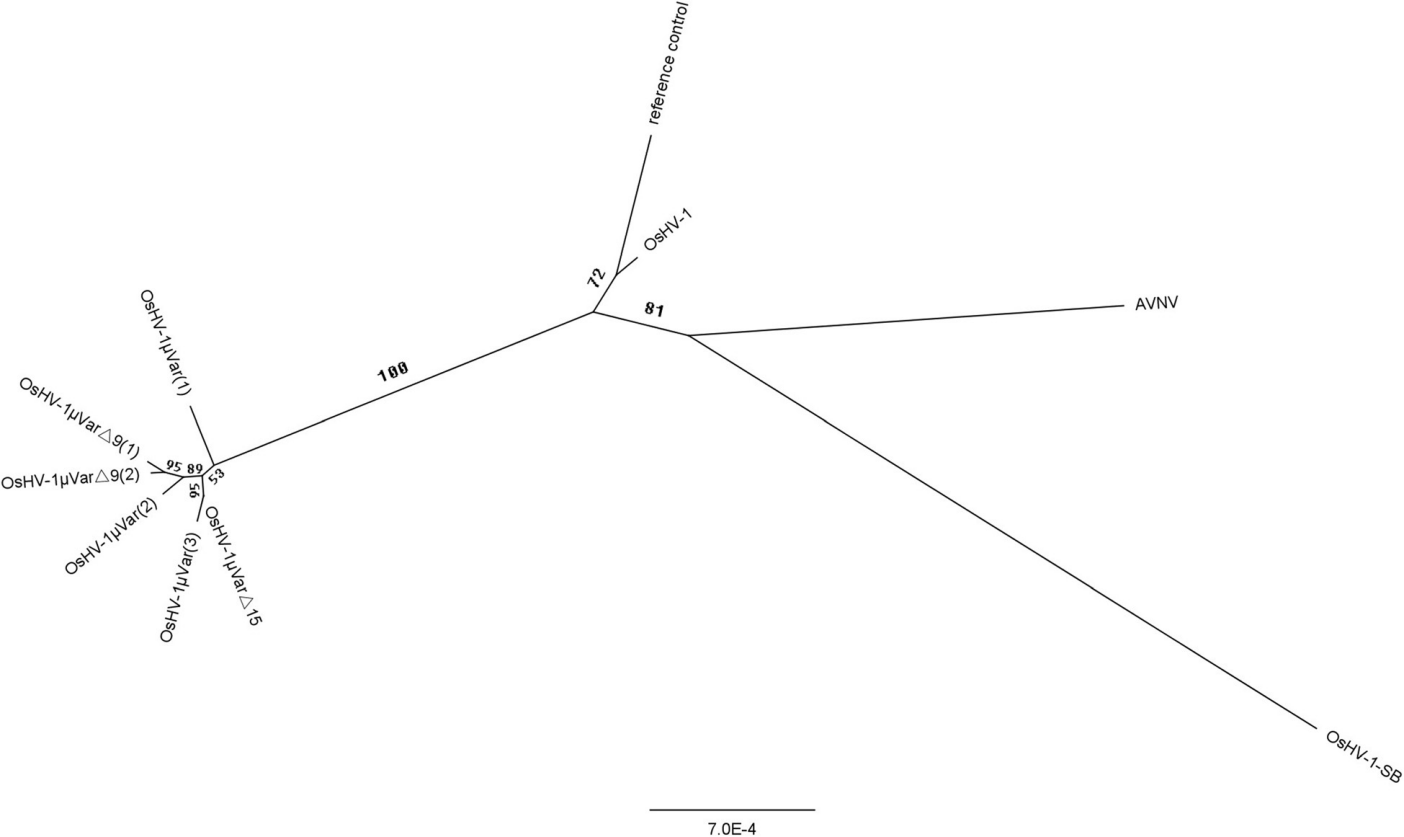
Bootstrap analysis (100 replicates) of unrooted phylogenetic trees of 32 ORFs constructed with the PAUP heuristic search algorithm. Numbers at the branches indicate bootstrap support value > 50 %

In order to study the relatedness between OsHV-SB and other variants of OsHV-1, phylogenetic trees were constructed from the nucleotides of 32 ORFs in ten variants of OsHV-1. The estimated phylogenetic tree divided the ten variants of OsHV-1 into two main groups with a bootstrap value of 100 % (Fig. 2). One group consisted of six closely related microvariants that identified in Europe after 2008. The second group was composed of the other four variants, which were subdivided into two clades containing OsHV-1-SB and AVNV, OsHV-1 and reference control respectively. The division of the four variants was bootstrap-supported over 70 % in both subclades. These results indicated OsHV-1-SB was more closely related to AVNV, a little further from OsHV-1 reference type, and the least related to the variant μVar and related ones. We presumed that the closer relationship between OsHV-1-SB and AVNV could be explained by their closer geographic distribution. A distant relationship between OsHV-1 reference type and the variant μVar and related ones was revealed here as found in the other studies [28, 33, 13], although both of them were found in France. But it is difficult to infer which factors have also contributed to the phylogenetic tree shape of these variants present here; further study is required.

Different epidemiological features have also been found in OsHV-1-SB and AVNV in China compared to OsHV-1 and OsHV-1 microvariants in Europe. High mortality rates of bivalves associated with infection of OsHV-1 and its microvariants in Europe were usually reported in *C.gigas* larvae and juveniles [2, 34, 3], although they could also be detected in asymptomatic adults with high prevalence [35–37]. While high mortalities of bivalves associated with infection of AVNV and OsHV-1-SB were usually found in adult bivalves [17].

## Conclusion

In this study, we have determined the whole genome sequence of a newly described OsHV-1 variant associated with the mass mortalities of broodstocks of *Scapharca broughtonii*. Through detailed comparison and analysis of the genome structure and sequences of different OsHV-1 variants, we found that OsHV-1-SB showed a number of variations compared to the other two OsHV-1 variants. Large indels and associated deletions and insertions of tens of ORFs were noticed, but further research is required to determine the function of these ORFs and their encoded proteins in relation to the pathogenicity of OsHV-1 to bivalves. Phylogenetic analysis based on the nucleotide sequences of 32 ORFs indicated that OsHV-1-SB is most closely related to AVNV.

## Methods

### Sample selection

Moribund blood clam adults with clinical signs including slow response, gaping valves and pale visceral mass were collected from hatcheries in Changdao, China in June 2012. Collected samples were conserved in ice box and transferred to laboratory immediately. The presence of virus DNA was then confirmed by PCR with C2/C6 primer pair [6]. Both the collection and handling of blood clams were conducted under the approval of the Animal Care and Ethics Committee, Yellow Sea Fisheries Research Institute, Chinese Academy of Fishery Science.

### Purification of virus and viral DNA

Virus particles were purified from moribund individuals as described by Ren et al. [19]. Viral DNA was extracted from purified virions with a TIANamp^TM^ Marine Animals DNA Kit (Tiangen Biotech, Beijing) according to the manufacturer’s protocol. The quality and concentration of the extracted DNA were determined by a micro volume spectrophotometer (Nanodrop 2000; Thermo Fisher Scientific Inc., West Palm Beach, FL, USA).

### PCR amplification and DNA sequencing

The genome sequence of OsHV-1-SB was determined using the primer walking approach. Briefly, 62 PCR primer pairs (Additional file 1: Table S2) designed based on the genome sequences of OsHV-1 reference type and AVNV were employed to amplify the whole genome sequence of OsHV-1-SB. The amplicons (from 698 to 4624 bp) were purified with TaKaRa gel purification kit (Takara), and then inserted into pEASYTM-T5 Zero Cloning Vector (Beijing TransGen Biotech). To exclude errors generated in the process of PCR and sequencing, three or more clones of each cloned fragment were sequenced in both directions with M13 forward and reverse primers with ABI PRISM 3770 (Shanghai Sunny Biotechnology Co., Ltd.). The genome termini were identified according to the method described by Ren et al. [19].

### Sequence analysis

Sequence assembly, genomic composition and structure were analyzed using DNASTAR 7.1 (DNASTAR Inc., USA). ORFs of OsHV-1-SB were predicted with NCBI ORF finder (http://www.ncbi.nlm.nih.gov/gorf/gorf.html) according to the criteria used in OsHV-1 reference type (Davison et al. [18]). Pairwise identities of putative amino acids shared among OsHV-1 reference type, AVNV and OsHV-1-SB were calculated with BLASTP (http://www.ncbi.nlm.nih.gov/) and MegAlign program (DNASTAR, Inc., USA). Initial alignment of OsHV-1-SB with OsHV-1 reference type and AVNV revealed that single nucleotide polymorphisms (SNPs) and insertion/deletion polymorphisms (indels) were spread across the genome. Therefore the search and characterization of small indels and SNPs were also carried out with Mega 5 [38]. Briefly, the nucleotide sequences of each ORF and non-coding regions were extracted manually from the three completed genome of each OsHV-1 variant. Then the number and size of small indels were obtained by aligning the homologous ORFs or non-coding regions of different OsHV-1 variants with ClustalW implanted in Mega 5. Finally, the number of SNP could be generated automatically by further exploring the aligned sequence data with Mega 5.

### Phylogenetic analysis

Phylogenetic relationships among the SB variant of OsHV-1, OsHV-1 reference type, AVNV and other variants of OsHV-1 were inferred on the basis of nucleotide sequences of 32 ORFs reported by Martenot et al. [28]. Nucleotide sequences of the ORFs were retrieved from Genbank, concatenated and aligned with those of OsHV-1-SB using the default settings in MAFFT version 7 [39, 40], followed by minor manual adjustments using BioEdit7.0.0 [41]. The best-fit nucleotide substitution model was determined using the Akaike Information Criterion (AIC) in jModelTest 2.1.4 [42, 43]. Phylogenetic analysis was performed using Maximum Likelihood (ML) with heuristic search implemented in the program PAUP* 4.0b10 [44]. The tree space was thoroughly sampled using 100 random sequence additions. Branch support was estimated with 1000 bootstrap replicates with 10 random sequence additions per bootstrap replication. Since no suitable out group could be found, the ML tree was displayed as unrooted.

### Nucleotide sequence accession number

The genome sequence has been submitted to GenBank under accession number KP412538.

## Additional file

Additional file 1: Table S1. Potential open reading frames of the OsHV-1-SB genome. **Table S2**. Primers used for genomic sequencing of OsHV-1-SB.

## Abbreviations

OsHV-1: Ostreid herpesvirus 1
OsHV-1-SB: SB variant of Ostreid herpesvirus 1
AVNV: Acute viral necrosis virus
ORF: Open reading frame
GP: Glycoprotein
bp: Base pair
kb: Kilobase pair
PCR: Polymerase chain reaction
