# Supplementary material for: Complete genome sequence of Ostreid herpesvirus-1 associated with mortalities of Scapharca broughtonii broodstocks
Source: Virol J. 2015 Jul 25;12:110. doi: 10.1186/s12985-015-0334-0 (PMC4514999; doi:10.1186/s12985-015-0334-0)
Supplement: Additional file 1: Table S1. — Potential open reading frames of the OsHV-1-SB genome. Table S2. Primers used for genomic sequencing of OsHV-1-SB. [file 12985_2015_334_MOESM1_ESM.docx]

Table S1 Potential open reading frames of the OsHV-1-SB genome.

| ORF | Position (nt) ^a^ | Length (aa) ^b^ | Predicted structure or function | OsHV-1 | | AVNV | |
| --- | --- | --- | --- | --- | --- | --- | --- |
|  |  |  |  | Identity (%) | Length(aa)^b^ | Identity (%) | Length (aa)^b^ |
| 1 | 114-560 | 148 |  | 99 | 148 | 98 | 148 |
| 2 | 679-1182 | 167 |  | 100 | 167 | 100 | 167 |
| 3 | 2051-2740 | 229 |  | 99 | 254 | 99 | 307 |
| 4.1 | 3462-4019 | 185 |  |  |  |  |  |
| 4.2 | 4125-4511 | 128 |  |  |  |  |  |
| 5^c^ | 4619-6420 |  |  |  |  |  |  |
| 6 | 6496-8526 | 676 |  | 99 | 676 | 100 | 676 |
| 7 | 8703-12248 | 1181 | Motifs V and VI characteristic of SF2 helicases | 99 | 1181 | 99 | 1181 |
| 8 | 12285-13244 | 319 |  | 99 | 319 | 99 | 319 |
| 9.1 | 13332-13757 | 141 |  |  |  |  |  |
| 9.2 | 13884-15110 | 408 | RING finger protein |  |  |  |  |
| 10 | 15370-16398 | 342 |  | 99 | 342 | 99 | 342 |
| 11 | 16665-19952 | 1095 |  | 99 | 1095 | 99 | 1095 |
| 12 | 20331-20645 | 104 |  | 100 | 195 | 100 | 195 |
| 13 | 20691-21008 | 105 |  | 100 | 105 | 100 | 105 |
| 14 | 21559-22110 | 183 |  | 99 | 194 | 99 | 194 |
| 15 | 22489-23133 | 214 |  | 100 | 214 | 99 | 214 |
| 16 | 23211-23426 | 71 | Membrane protein | 99 | 76 | 99 | 71 |
| 17 | 23699-23941 | 80 | secreted protein | 99 | 80 | 99 | 80 |
| 18 | 23993-24274 | 93 |  | 99 | 93 | 64 | 58 |
| 19 | 24321-25529 | 402 |  | 99 | 402 | 99 | 402 |
| 20 | 25609-27348 | 579 | Ribonucleotide reductase small subunit | 99 | 579 | 100 | 579 |
| 21 | 27419-30376 | 985 |  | 99 | 984 | 97 | 669 |
| 22 | 30541-35439 | 1632 |  | 99 | 1632 | 99 | 1632 |
| 23 | 35571-39389 | 1272 |  | 99 | 1272 | 99 | 1272 |
| 24 | 39486-40619 | 377 | Primase | 99 | 377 | 99 | 350 |
| 25 | 40702-41367 | 221 | Class I membrane protein | 99 | 221 | 99 | 221 |
| 26 | 41415-42602 | 395 |  | 99 | 395 | 99 | 395 |
| 27 | 42711-43511 | 266 | Inactive dUTPase | 99 | 266 | 99 | 266 |
| 28 | 43596-46157 | 853 |  | 99 | 853 | 99 | 951 |
| 29 | 45847-46452 | 201 |  | 98 | 201 |  |  |
| 30 | 46555-47301 | 248 | Related via cysteine-rich domain to ORF31 of genus Rhadinovirus and UL92 of genus Cytomegalovirus of the Herpesviridae | 100 | 248 | 100 | 248 |
| 31 | 47355-47912 | 185 |  | 99 | 185 | 99 | 185 |
| 32^c^ | 48173-50368 |  | Encodes class I membrane protein |  |  |  |  |
| 33 | 50380-51270 | 296 |  | 99 | 296 | 99 | 296 |
| 34 | 51350-51724 | 124 | Inactive dUTPase | 99 | 124 | 99 | 124 |
| 35 | 51810-52283 | 157 |  | 100 | 157 | 100 | 157 |
| 36 | 52353-52580 | 75 | Membrane protein | 100 | 75 | 100 | 75 |
| 37 | 52650-53276 | 208 | RING finger protein |  |  |  |  |
| 39 | 53521-54105 | 194 |  | 99 | 194 | 100 | 194 |
| 40 | 54108-55835 | 575 |  | 100 | 575 | 99 | 575 |
| 41 | 55985-58903 | 972 |  | 99 | 972 | 99 | 972 |
| 42 | 58942-60036 | 364 | BIR protein containing RING finger | 99 | 364 | 99 | 364 |
| 43 | 60155-60766 | 203 |  | 99 | 203 | 99 | 203 |
| 125 | 60824-61771 | 315 |  |  |  |  |  |
| 126 | 62063-62710 | 215 |  |  |  |  |  |
| 127 | 62897-63712 | 271 |  |  |  |  |  |
| 44 | 63785-64711 | 308 |  | 99 | 308 | 99 | 308 |
| 45 | 64854-65579 | 241 |  | 99 | 241 | 99 | 241 |
| 46 | 65727-66374 | 215 |  | 99 | 215 | 99 | 215 |
| 47 | 66390-70628 | 1412 |  | 99 | 1412 | 99 | 1412 |
| 49 | 70834-74250 | 1138 | Contains motifs V and VI characteristic of SF2 helicases | 99 | 1138 | 99 | 1138 |
| 51 | 75569-78076 | 835 | Ribonucleotide reductase large subunit | 99 | 835 | 99 | 835 |
| 52 | 78193-78735 | 180 |  | 100 | 180 | 100 | 180 |
| 53 | 78819-80366 | 515 | RING finger-like protein | 99 | 515 | 99 | 515 |
| 54 | 80425-82848 | 807 | Class I membrane glycoprotein | 99 | 807 | 99 | 807 |
| 55 | 82925-83344 | 139 |  | 100 | 139 | 99 | 139 |
| 56 | 83617-84465 | 282 |  | 99 | 282 | 99 | 282 |
| 57 | 84215-85165 | 316 | Multiple transmembrane protein; chloride channel | 100 | 316 | 99 | 316 |
| 58 | 85211-86776 | 521 |  | 99 | 521 | 99 | 521 |
| 59 | 86869-90111 | 1080 | Class I membrane protein | 99 | 1080 | 99 | 1080 |
| 60 | 90164-91399 | 411 |  | 99 | 411 | 99 | 411 |
| 61 | 91577-93313 | 578 |  | 100 | 578 | 97 | 319 |
| 62^c^ | 93472-95272 |  |  |  |  |  |  |
| 63^c^ | 95378-97241 |  |  |  |  |  |  |
| 64 | 97281-98033 | 250 | RNA ligase | 99 | 398 | 97 | 250 |
| 65^c^ | 98585-100569 |  |  |  |  |  |  |
| 66 | 100624-104016 | 1130 |  | 99 | 1130 | 99 | 1130 |
| 67 | 104430-106220 | 596 | SF2 helicase | 99 | 596 | 99 | 596 |
| 68 | 106300-108390 | 696 | Class I membrane protein | 99 | 693 | 99 | 694 |
| 69 | 108458-109849 | 463 |  | 99 | 463 | 99 | 463 |
| 70 | 109920-110933 | 337 |  | 82 | 200 | 82 | 200 |
| 71 | 111220-112581 | 453 |  | 99 | 453 | 99 | 453 |
| 72 | 112479-113045 | 188 | Membrane protein | 100 | 188 | 99 | 188 |
| 73^c^ | 113266-114945 |  |  |  |  |  |  |
| 74 | 115043-115399 | 118 |  | 100 | 118 | 100 | 118 |
| 75 | 115455-116165 | 236 | dUTPase | 100 | 236 | 99 | 247 |
| 76 | 116637-118673 | 678 |  | 99 | 678 | 99 | 678 |
| 77 | 118791-122585 | 1264 | Class I membrane protein | 99 | 1264 | 99 | 1263 |
| 78 | 122870-126094 | 1074 |  | 99 | 1151 | 99 | 1074 |
| 79 | 126109-126549 | 146 |  | 100 | 146 | 99 | 146 |
| 80 | 126613-126963 | 116 | Membrane protein | 99 | 116 | 99 | 116 |
| 81 | 127086-127727 | 213 |  | 100 | 213 | 100 | 213 |
| 82 | 127675-128565 | 296 |  | 98 | 296 | 98 | 296 |
| 83 | 128660-129766 | 368 |  | 99 | 368 | 99 | 368 |
| 84 | 129772-130128 | 118 | Membrane protein | 100 | 118 | 99 | 118 |
| 85 | 130133-132136 | 667 |  | 99 | 667 | 99 | 667 |
| 86 | 132140-132547 | 135 |  | 100 | 135 | 99 | 135 |
| 87 | 132562-133074 | 170 | BIR protein lacking RING finger | 99 | 170 | 99 | 170 |
| 88 | 133167-135413 | 748 | Class I membrane protein | 99 | 748 | 99 | 748 |
| 89 | 135465-136199 | 244 |  | 99 | 244 | 99 | 244 |
| 90 | 136260-137144 | 294 |  | 99 | 294 | 99 | 294 |
| 91 | 137261-138343 | 360 |  | 100 | 360 | 100 | 360 |
| 92 | 138392-139081 | 229 |  | 99 | 229 | 99 | 229 |
| 93 | 139017-140231 | 404 |  | 99 | 404 | 99 | 404 |
| 94 | 140236-141279 | 347 |  | 99 | 347 | 99 | 347 |
| 95 | 141272-142297 | 341 | Similar to a family of uncharacterized, conserved eukaryotic proteins | 99 | 310 | 99 | 341 |
| 96 | 142363-143085 | 240 | RING finger protein | 100 | 240 | 100 | 240 |
| 97 | 143175-143720 | 181 | RING finger protein | 100 | 181 | 99 | 181 |
| 98 | 144223-145977 | 547 |  | 99 | 547 | 98 | 547 |
| 99 | 146346-147098 | 250 | BIR protein lacking RING finger | 99 | 250 | 99 | 250 |
| 100 | 147589-153225 | 1878 | DNA polymerase | 99 | 1878 | 99 | 1878 |
| 101 | 153312-153941 | 209 |  | 99 | 209 | 99 | 209 |
| 102 | 153982-156270 | 762 |  | 99 | 762 | 99 | 762 |
| 103 | 156283-157554 | 423 | Multiple transmembrane protein | 99 | 423 | 99 | 176 |
| 104 | 157702-161313 | 1203 |  | 99 | 1203 | 99 | 1200 |
| 105^c^ | 161450-163060 |  |  |  |  |  |  |
| 106 | 163205-164077 | 290 | BIR protein containing RING finger | 97 | 465 | 97 | 464 |
| 107 | 164735-166804 | 689 |  | 99 | 689 | 99 | 689 |
| 108 | 167020-167832 | 270 |  | 99 | 270 | 99 | 270 |
| 109 | 167885-170509 | 874 | ATPase subunit of DNA-packaging terminase | 100 | 874 | 100 | 874 |
| 110 | 170604-171389 | 261 |  | 99 | 261 | 99 | 261 |
| 111 | 171479-172348 | 289 | Multiple transmembrane protein | 100 | 289 | 99 | 289 |
| 112 | 172472-173860 | 462 |  | 99 | 462 | 99 | 462 |
| 113 | 173867-174823 | 318 |  | 99 | 318 | 99 | 318 |
| 114 | 175046-176233 | 395 |  | 43 | 494 | 43 | 494 |
| 3 | 177069-177758 | 229 |  | 99 | 254 | 99 | 307 |
| 2 | 178626-179129 | 167 |  | 100 | 167 | 100 | 167 |
| 1 | 179248-179694 | 148 |  | 99 | 148 | 98 | 148 |
| 116 | 180868-181638 | 256 |  | 99 | 256 | 96 | 250 |
| 118 | 182520-183188 | 222 | RING finger protein | 98 | 222 | 98 | 222 |
| 119 | 183557-184315 | 252 |  | 99 | 192 | 100 | 252 |
| 120 | 184579-184908 | 109 |  | 99 | 76 | 98 | 109 |
| 121 | 186069-186716 | 215 | RING finger protein | 98 | 215 | 100 | 215 |
| 124 | 187530-188954 | 474 | RING finger-like protein | 97 | 474 | 97 | 474 |
| 122 | 190596-191750 | 384 |  | 99 | 384 | 99 | 384 |
| 121 | 192451-193098 | 215 |  | 98 | 215 | 100 | 215 |
| 120 | 194259-194588 | 109 |  | 99 | 76 | 100 | 109 |
| 119 | 194850-195608 | 252 |  | 99 | 192 | 100 | 252 |
| 118 | 195977-196645 | 222 | RING finger protein | 99 | 222 | 100 | 222 |
| 116 | 197527-198297 | 256 |  | 99 | 256 | 97 | 250 |

^a^ indicated the positions of the ORFs in the genome

^b^ indicated the lengths of amino acid residues encoded by the ORFs

^c^ indicated disrupted genes which contain more than one small ORFs

Table S2. Primers used for genomic sequencing of OsHV-1-SB.

| Primer name | Sequence(5’-3’) | Size (bp) |
| --- | --- | --- |
| 1F | TCCCGCCAATACCCATAATGCA^a^ | 3468 |
| 1R | TGTGCCCGTTAGACTGGGAAAG^a^ |  |
| 2F | CACGCCTCTTTCCCAGTCTAAC^a^ | 3149 |
| 2R | AGGAGCCAGACCAAAACCATTC ^a^ |  |
| 3F | AATTGGGATGCGATCGTTTCCTG^a^ | 3874 |
| 3R | TGGACTTTGAGGCTCCCATTTGT ^a^ |  |
| 4F | AGGAAGGCAACAGGATAGAAATG ^a^ | 3340 |
| 4R | ACAAGGAGGTTGGAAGTCGTAGA^a^ |  |
| 5F | TGGATAAATGACCGGCGAGAAAG^a^ | 3318 |
| 5R | TGAAATCACCAACACTGGAGGCT^a^ |  |
| 6F | AAGGATCTGTCTAAGCCTCCAGT^a^ | 3735 |
| 6R | CTCGTTGAAACATCTACCGTCAT ^a^ |  |
| 7F | TTAGGTATGCCAGCCAGAGTTCA^a^ | 3222 |
| 7R | CTTCCACCCGGAGAAAGGATTAA^a^ |  |
| 8F | TATGAACATGCCGCTTCTACTTC ^a^ | 3195 |
| 8R | CAATGCCTATAAAGACCCTGAA^a^ |  |
| 9F | TTTATCGGTGCAAATTCAGGGTC^a^ | 3132 |
| 9R | CTAAATGGTCGCCTCAAGGTTGG^a^ |  |
| 10F | CATACAATAGTTGGGTGGTCAA^a^ | 3677 |
| 10R | GATGATGTGGGTTCTGGTCTAA^a^ |  |
| 11F | AGGAAATTAACACCCGAGACAA^a^ | 3680 |
| 11R | AGCACAAACACTACCGCATACA^a^ |  |
| 12F | TCTGTTCATGTATGCGGTAGTGT^a^ | 2912 |
| 12R | ATAGCCTGCCGATATTCCTTGC^a^ |  |
| 13F | TTAGTATGGCAAGGAATATCGG^a^ | 3132 |
| 13R | AAGGTGGTTCTGGAGTTGAGAC^a^ |  |
| 14F | CAGGGTGATTGTCTCAACTCCAG^a^ | 3008 |
| 14R | ACCCGACAGTGAACTCCCTACTC^a^ |  |
| 15F | ATGTAAATCCAAACCCTGTGCGT^a^ | 3533 |
| 15R | GTAGTTTCATCGGTTCCAGCCTC^a^ |  |
| 16F | ATATTCTATAATCCCAGGAGCG^a^ | 3546 |
| 16R | GATGTAACCAATGTACCACCGT^a^ |  |
| 17F | CTGAACGGTGGTACATTGGTTAC^a^ | 3393 |
| 17R | CCTATTCTCCCGGTCGTGATACA^a^ |  |
| 18F | GTATCACGACCGGGAGAATAGG^a^ | 3260 |
| 18R | TTTACGAAACTGGGAGACCACA^a^ |  |
| 19F | CATGTTCGCTACTTGGAATGTC^a^ | 3584 |
| 19R | AGGAGGACATCTGGCGGTA |  |
| 20F | CGTGCTGAGACGGAATGTG | 3275 |
| 20R | TGTTGACTACGCCAATGACT |  |
| 21F | AAGCGTCTCATCGAACTACGGG^a^ | 3764 |
| 21R | ATGTGGTCACCTTTGTGCCTGT^a^ |  |
| 22F | TGGATGTGGTTTTGGATACGAA^a^ | 3770 |
| 22R | AGTGGTGGTGGCTCTTACTCTG^a^ |  |
| 23F | CACCGCAAATCTCCGATATGAT^a^ | 2139 |
| 23R | ATGAATTTCCAAGATAAAGATATCGGGA |  |
| 24F | CCTTTCATTCACGGCATCCT | 2366 |
| 24R | TCCACTCGGAACCCCTGAGATA^a^ |  |
| 25F | ATTGCACAATATCTCAGGGGTTC^a^ | 4624 |
| 25R | ATCCACCATAGATTGCGTCAGT^a^ |  |
| 26F | GTGAAGAAACACTGACGCAATC^a^ | 3975 |
| 26R | AATAATCCTCGGGACCCAAGTT^a^ |  |
| 27F | ACCGGGTGACCCAGTGTTTAA^a^ | 3131 |
| 27R | GGTGGATTCATGTGCGATCAT^a^ |  |
| 28F | CTGTGATCTTGTCGAACAATTC^a^ | 4078 |
| 28R | TTCGGTCATGTACTCTATCTCCTG^a^ |  |
| 29F | CTGTGGGATGTATAGCCGTGGAG^a^ | 3720 |
| 29R | CCTGATTGGAGGGCAGAATGTA^a^ |  |
| 30F | GAAGTTGACTTTCTTTCCCTTGC^a^ | 3925 |
| 30R | ACTGAGGTTGTGGGGTGTATTGT^a^ |  |
| 31F | TTTTCCCAGCAAACAGACCTAT^a^ | 4456 |
| 31R | GACATACCCAGTATTGCACCTT^a^ |  |
| 32F | TCTACACTCTGAGACCATTCCT^a^ | 4120 |
| 32R | GATTTGTACCCAGACCAGTCAT^a^ |  |
| 33F | AGGTTTCGGCGAGTCGATGA^a^ | 3555 |
| 33R | ATGGCTTGCTGTTTTACACGTTCT^a^ |  |
| 34F | GAGACGCATTAAAATGATGGAAG^a^ | 3113 |
| 34R | ACAGTCTGGAAATTCTCGTAGCC^a^ |  |
| 35F | CGGGCTACGAGAATTTCCAGAC^a^ | 3731 |
| 35R | CGATGATTCAGACAGGCAAGCT^a^ |  |
| 36F | ACCAACAGCTTGCCTGTCTGAA^a^ | 3596 |
| 36R | GGAATGTCGCTTTGTACCACTG^a^ |  |
| 37F | CAGAACCTGTAATAGCAAACGA^a^ | 4123 |
| 37R | GGGAGATGTACGACGATGTGAA^a^ |  |
| A3 | GCCAACCGTTGGAACCATAACA^b^ | 1001 |
| A4 | GGGAATGAGGTGAACGAAACTATAGACC^b^ |  |
| 39F | TTTGTCCGAGAATTTGGTATCC^a^ | 4369 |
| 39R | AACACCGACCATGAATCTAGGG^a^ |  |
| Gp3 | GGTTGTGGGTTTGGAAATGTAGA^c^ | 698 |
| Gp4 | GGCGTCCAAACTCGATTAAA^c^ |  |
| 41F | GCCGGTGAATGGATGTTATGTC^a^ | 3158 |
| 41R | TAACCACGCTGGCTACGACTAA^a^ |  |
| 42F | CGCCTTTACCATTCATAACAAG^a^ | 3050 |
| 42R | CCAGCATTACTGACATCCCTTA^a^ |  |
| 43F | AACCGAGGTGTAAGGGATGTCA^a^ | 3678 |
| 43R | GATGCTCCGTGCTTGAGCCTAT^a^ |  |
| 44F | CCCGTTATTAGTCATCAGCTCC^a^ | 3570 |
| 44R | TCAACGAGCAATCCCCTTTATA^a^ |  |
| 45F | CCGAAAATCTGATTGGCTGCTAT^a^ | 2952 |
| 45R | GACACCTGGGTATTGCGAGT |  |
| 46F | TTTGTTCACCAGACCCTTCC | 3195 |
| 46R | ATCCTTGGTGGAGTGTATAACC^a^ |  |
| 47F | ACAAAACTGCGGTTATACACTC^a^ | 3251 |
| 47R | TCTTCGCCTACATATTTACTGG^a^ |  |
| 48F | GAAGATGAAGAGGAAGACGAAAAG^a^ | 3460 |
| 48R | TGGGGAATACAACGTGAGATAA^a^ |  |
| 49F | GCTGATTAAGATTGGCAAAGAA^a^ | 2583 |
| 49R | AACACGCTAGATATGGTCGGTA^a^ |  |
| 50F | TACCGACCATATCTAGCGTGTT^a^ | 3069 |
| 50R | GGGTAACGGTTTATCATTCACA^a^ |  |
| 51F | AGGATGATGTGAATGATAAACCG^a^ | 2512 |
| 51R | TGCTTTGCTCAAAACCCTCTATA^a^ |  |
| 52F | TTTATCTGATGCCGACAGAGTCA^a^ | 2927 |
| 52R | TCATCTTCAAAAGCGTCGATG^a^ |  |
| 53F | CAGCAAAGTTTACATCGACGCTT^a^ | 3998 |
| 53R | GCGCTTTCGCATTCCACATC |  |
| ORF114For | GGTTGGATCTCTTGGGAATGG^d^ | 2742 |
| 54R | AAGATAGGCGACACGGGACT |  |
| 55F | TGGCAACGTCCCTATCGTAA | 2191 |
| 55R | ACTTCGTGTTGTTGTTGAGCCAT^a^ |  |
| 56F | GAGAAGAAGCAGCAACAGGTTAA^a^ | 2165 |
| 56R | TTGTGGGTGTATCTGCTAGTGTC^a^ |  |
| 57F | ACAGGGATTACGTGTACCACAG^a^ | 2400 |
| 57R | GCATAGGATAGCCATCAGCAAG^a^ |  |
| 58F | GATCCAATCACAGGCGATACCT^a^ | 2550 |
| 58R | ACAGTTTGGTGGAGGAGGTG |  |
| ORF121Rev | ACATCCAATGAAAACAGCCGGAA^d^ | 2093 |
| 59R | GGCGGGGAAAATAAGTCCTG |  |
| 60F | ACCACTTAACCCACAACCATAT^a^ | 4449 |
| 60R | AAGACTCCCAAGCAAGTTATGA^a^ |  |
| 61F | GCGCTATTGCCCATGTTAAAAT^a^ | 3133 |
| 61R | AGGCGATACCTTGCTGATGG |  |
| 62F | CCATTCATTCTCCAATCCCTC | 3984 |
| 62R | GGGGCGTGTCTAACCTCCTA |  |

^a^: Weicheng Ren. Detection methods, sequence of the complete genome of acute viral necrosis virus Isolated from Scallop, *Chlamys farreri.* PhD dissertation, Ocean University of China, Qingdao, China; 2009.

^b^: Renault T, Lipart C, Arzul I. A herpes-like virus infecting Crassostrea gigas and Ruditapes philippinarum larvae in France. J Fish Dis. 2001;24(6):369-76.

^c^: Arzul I, Nicolas JL, Davison AJ, Renault T. French scallops: A new host for Ostreid herpesvirus-1. Virology. 2001;290(2):342-9.

^d^: Martenot C, Travaille E, Lethuillier O, Lelong C, Houssin M. Genome exploration of six variants of the Ostreid Herpesvirus 1 and characterization of large deletion in OsHV-1 mu Var specimens. Virus Res. 2013;178(2):462-70.
